# Motion artifact variability in biomagnetic wearable devices

**DOI:** 10.3389/fmedt.2024.1457535

**Published:** 2024-10-17

**Authors:** Negin Ghahremani Arekhloo, Huxi Wang, Hossein Parvizi, Asfand Tanwear, Siming Zuo, Michael McKinlay, Carlos Garcia Nuñez, Kianoush Nazarpour, Hadi Heidari

**Affiliations:** ^1^ Neuranics Limited, Glasgow, United Kingdom; ^2^ Microelectronics Lab, James Watt School of Engineering, University of Glasgow, Glasgow, United Kingdom; ^3^ School of Informatics, The University of Edinburgh, Edinburgh, United Kingdom

**Keywords:** biomagnetic measurements, motion artifacts, wearable sensors, gradient background field, homogeneous background field

## Abstract

Motion artifacts can be a significant noise source in biomagnetic measurements when magnetic sensors are not separated from the signal source. In ambient environments, motion artifacts can be up to ten times stronger than the desired signals, varying with environmental conditions. This study evaluates the variability of these artifacts and the effectiveness of a gradiometer in reducing them in such settings. To achieve these objectives, we first measured the single channel output in varying magnetic field conditions to observe the effect of homogeneous and gradient background fields. Our analysis revealed that the variability in motion artifact within an ambient environment is primarily influenced by the gradient magnetic field rather than the homogeneous one. Subsequently, we configured a gradiometer in parallel and vertical alignment with the direction of vibration (X-axis). Our findings indicated that in a gradient background magnetic field ranging from 1 nT/mm to 10 nT/mm, the single-channel sensor output exhibited a change of 164.97 pT per mm unit increase, while the gradiometer output showed a change of only 0.75 pT/mm within the same range. Upon repositioning the gradiometer vertically (Y direction), perpendicular to the direction of vibration, the single-channel output slope increased to 196.85 pT, whereas the gradiometer output only increased by 1.06 pT/mm for the same range. Our findings highlight the influence of ambient environments on motion artifacts and demonstrate the potential of gradiometers to mitigate these effects. In the future, we plan to record biomagnetic signals both inside and outside the shielded room to compare the efficacy of different gradiometer designs under varying environmental conditions.

## 1 Introduction

The movement of charged ions, such as Na^+^, K^+^, and Ca^2+^, across the membrane of the excitable cell generates electrical potentials on the body surface. These electrical signals can be recorded through various methods, including Electromyography (EMG) from skeletal muscle, Electrocardiography (ECG) from the heart, and Electroencephalography (EEG) from the brain. According to Maxwell's equation on electromagnetism, time-varying electric and magnetic fields are inherently linked; thus, whenever there are electric fields, there are also magnetic fields, and vice versa. Consequently, the same ion fluxes that generate electrical currents also produce magnetic fields which can be measured either electrically or magnetically (1). These measurable magnetic fields are referred to as biomagnetism or bioelectromagnetism, encompassing techniques such as magnetomyography (MMG) from skeletal muscle, magnetocardiography (MCG) from the heart, and magnetoencephalography (MEG) from the brain (2).

**Figure 1:**
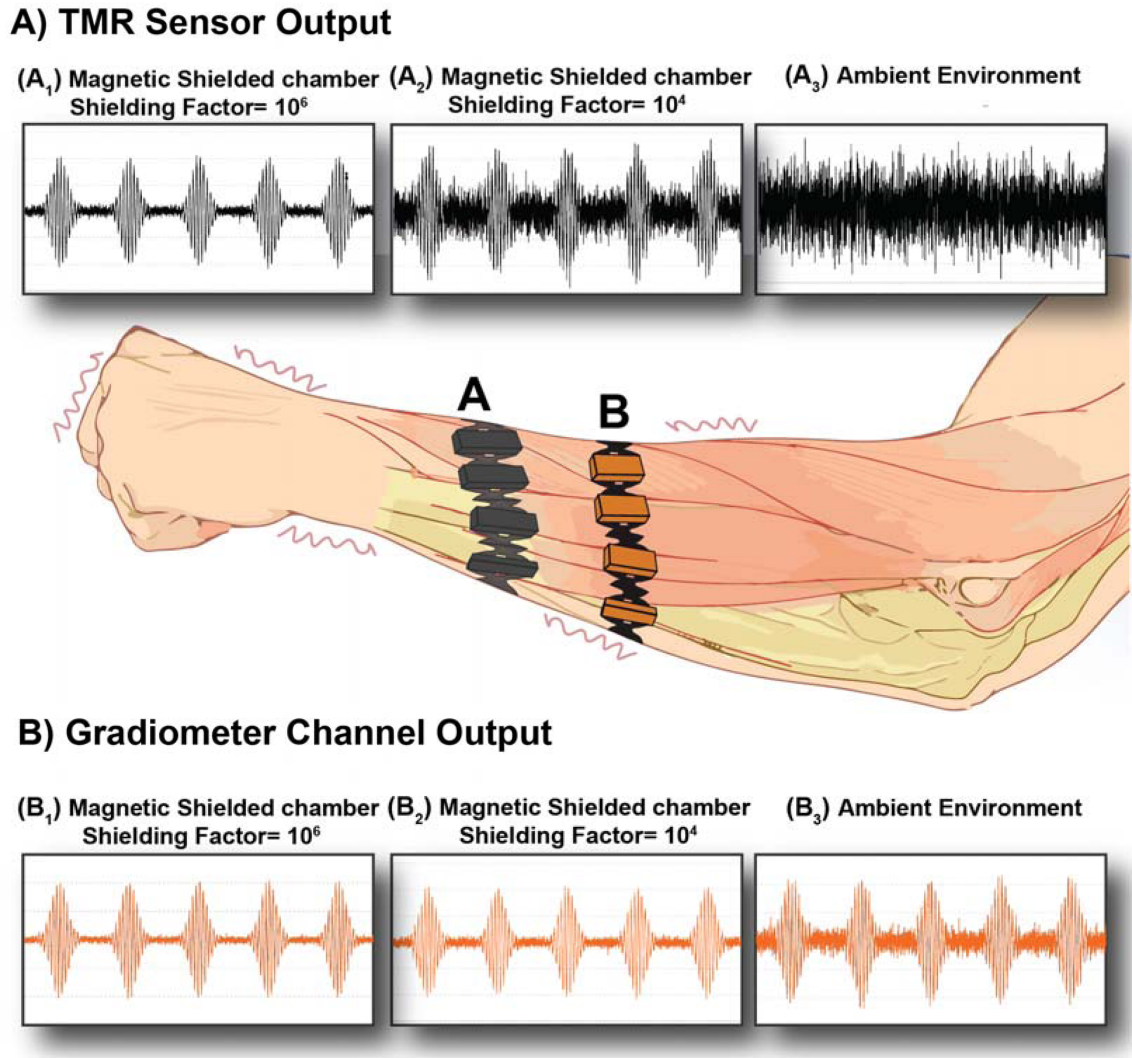
Motion/vibration artifacts in wearable MMG with TMR sensors and gradiometer. **(A)** Signals detected through TMR sensors. **(A1)** MMG signals detected in a highly shielded environment (10^6^) showing minimal motion artifacts. **(A2)** MMG signals detected in a less shielded environment (10^4^), exhibiting higher levels of motion artifacts, although the desired signals remain detectable. **(A3)** MMG signals detected in an ambient environment, where motion artifacts significantly pollute the signals, making the target signals undetectable. **(B)** Signals detected through gradiometer. **(B1)** MMG signals detected in a highly shielded environment (10^6^) showing minimal motion artifacts. **(B2)** MMG signals detected in a less shielded environment (10^4^), exhibiting a comparable level of motion artifacts to **B1**. **(B3)** MMG signals are detected in an ambient environment where the target signals are still detectable. Plots (**A1–3** and **B1–3**) are schematic representations based on the literature on EMG and MMG (9–11).

While magnetic measurements are typically conducted within magnetically shielded rooms, recent advancements in sensor technology have enabled some MCG or MEG measurements to be performed in an ambient environment, through increased dynamic range and reduced system noise (3, 4). In such settings, especially if the sensor is not separated from the signal source, one challenge will be the presence of fluctuating motion artifacts, which constitute a significant noise source (5–8). As shown in Figure 1A, the magnitude of these artifacts varies depending on the surrounding environment, with different environments potentially altering the magnitude of the motion artifacts. For instance, in a shielded environment with a high shielding factor (10^6^), the motion artifact is minimal. When the shielding factor decreases to 10^4^, the motion artifact increases, but the desired signals remain detectable, as illustrated in Figures 1A1,A2. In contrast, in an ambient environment (Figure 1A3), the noise level is so high that the signal cannot be detected. This variability adds complexity to biomagnetic measurements and signal classification, as artifacts can fluctuate up to ten times greater than the measured signal, depending on the ambient magnetic field (12).

Several strategies exist to mitigate motion artifacts, including high-pass filters at 20 Hz for electrical signals. However, a higher cut-off frequency may be applied for magnetic signals since their frequency does not attenuate as much as electrical signals (13). Another approach is separating the sensor from the skin surface, reducing noise but significantly diminishing signal magnitude as the magnetic signal decreases by 1/r^3^, where r is the distance between the signal source and the sensor (14, 15). However, due to certain limitations, these mitigation strategies may not always be feasible. In MCG measurements, when the sensor is placed on the chest surface, breathing artifacts and chest microvibrations, caused by heart contractions and blood ejection into the vascular tree, contaminate MCG signals. Since their frequency ranges from 0.8 to 30 Hz, they overlap spectrally with the genuine MCG signals (16–19). Therefore, these artifacts cannot be effectively eliminated through signal filtering methods like cubic-spline interpolation (CSI), empirical mode decomposition (EMD) and wavelet (WAV) filtering, as these filtering methods are not only frequency-dependent but can also cause errors in the ST segment of the MCG, which contains diagnostic information associated with ischemic heart diseases (20–23). Therefore, the filtering methods will remove both the interference and part of the heart signals.

In MMG measurements, while the frequencies of interest typically lie in a higher frequency bandwidth than MCG, activities such as running can increase the frequency of motion artifacts, potentially causing overlap with the low-frequency content of genuine muscle activity (19, 24). In these conditions, filtering also may not be helpful. Additionally, in MMG, applying a gap between the muscle and the sensor can add complexity to signal analysis. Firstly, skeletal muscles move during contraction and relaxation, changing the distance between the sensor and the skin, affecting the signal amplitude and frequency (25). Moreover, muscle volume, as a critical indicator, influences the sensor-source distance effect on signal changes. For example, the muscle volume in different skeletal muscles, such as the Biceps Brachii muscle with a large volume vs. the Abductor Pollicis Brevis with a minor muscle volume, should be considered to assess the rate of changes in recorded MMG signals at various source-skin distances (26).

Furthermore, in the design of wearable biomagnetic devices, utilising high-pass filters is impractical, and creating a gap between the sensor and the skin surface is also impossible. Therefore, the influence of the environmental context on artifact magnitude must be considered. These considerations are paramount to ensuring the reliability of biomagnetic measurements in dynamic and varied conditions.

To develop a practical approach to avoid motion artifacts, it is essential first to gain a comprehensive understanding of their fundamentals in biomagnetism—a task that, to the best of our knowledge, has not yet been undertaken. Delving into the basics of motion/vibration artifacts will provide insights that could lead to innovative solutions, enabling more accurate biomagnetic measurements across diverse environments and dynamic conditions. Therefore, in this paper, we first aim to gain a comprehensive understanding of the basics of motion artifacts in biomagnetic measurements and then recommend using a gradiometer to eliminate the effects of these artifacts. As illustrated in Figure 1B, we hypothesize that the use of a gradiometer can eliminate the impact of vibration artifacts in various environments, ranging from a highly shielded magnetic chamber (Figure 1B1) to a less shielded chamber (Figure 1B2) and an ambient environment (Figure 1B3). This paper will also evaluate the efficacy of the gradiometer method to demonstrate its potential to improve the accuracy of biomagnetic measurements.

## 2 Methodology

### 2.1 Magnetised linear motor

A magnetised linear motor (DM01–23 × 80F-HP-R-100_MS13, Quinn Systems) was employed to generate controlled vibratory motion to simulate the artifact. The motor was selected for its capability to precisely modulate movement parameters such as velocity, distance, and acceleration/deceleration. For our experiment, we set the velocity to 1 m/s, with acceleration and deceleration at 0.5 m/s^2^, with the total travel distance fixed at 0.5 mm. To determine the frequency of the motor under these specified conditions, we attached a triboelectric nanogenerator (TENG) sensor to the end of the sensor mount. The TENG was constructed with a Zinc oxide (ZnO) thin film layered onto Aluminium foil, and Polyethylene terephthalate film, Indium Tin Oxide coated (PET-ITO) (with a surface resistivity of 60 Ω/sq, dimensions 1 ft ×1 ft × 5 mil, Sigma Aldrich Co Ltd, 639303) was positioned at the motor's final stop (27). The voltage output during contact was recorded using an oscilloscope (Keysight DSOX2012A) connected via a 100M*Ω* input impedance probe (model BKPR2000B-ND, B&K Precision). The oscilloscope data were then processed through a peak detection algorithm to establish the motor's operating frequency. The calculated frequency of the set parameters was 8 Hz.

### 2.2 Magnetically shielded chamber

**Figure 2:**
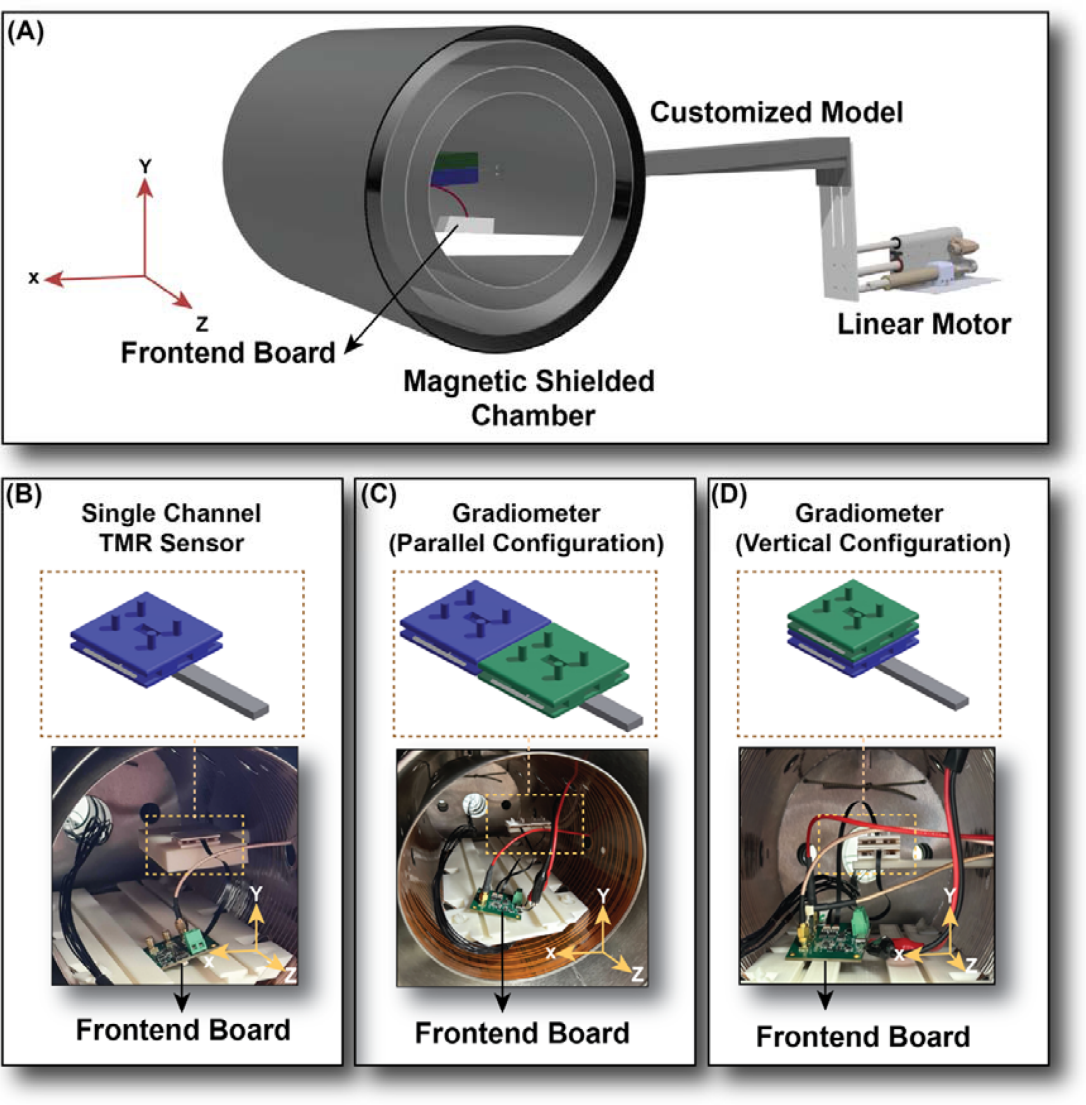
Experiment setup. **(A)** The 3D schematic design of the printed customised model, Magnetised linear motor, Twinleaf-MS2 magnetically shielded chamber, and frontend board, located separately from the sensor part, to avoid any interference from the frontend vibration on the sensor output. **(B)** Single channel TMR sensor inside the chamber, placed on the white 3D printed supporter coming in from one of the holes on the chamber wall. The sensor and the frontend are placed separately to avoid interference from the frontend vibration. **(C)** Gradiometer (Parallel configuration) with two sensors placed beside one another with the baseline in the X direction. **(D)** Gradiometer (Vertical configuration) with two sensors placed on top of one another with the baseline in the Y direction.

Considering the substantial influence of environmental conditions on the magnitude of motion artifact, a magnetically shielded chamber (Twinleaf-MS2) was utilised to establish a controlled experimental setting, as shown in Figure 2A (28). This chamber provides four layers high-permeability metal shielding designed to significantly attenuate ambient magnetic noise originating from electronic devices or other laboratory equipment, which could otherwise impact the magnetic measurements. Additionally, it features internal coils with a diameter of 180 mm and a length of 360 mm, allowing the precise alteration of the magnetic field within the centre of the shielded chamber. The amplitude of the current inside the coils needed to achieve the target magnetic field magnitude, known as the conversion factor, is 56.5 nT/mA for homogeneous magnetic fields and 1.82 nT/cm/mA for gradient magnetic fields, respectively. The magnetically shielded chamber was placed on a separated damped optical table to decouple any mechanical vibrations from the motor to the chamber. The distance between the motor core and chamber centre is 710 mm to minimize interference from the motor's internal magnets.

### 2.3 Experimental setup

We designed a custom 3D setup, as shown in Figure 2A, engineered to transmit vibrations from the motor to the sensor within the magnetically shielded chamber. The construction material for the 3D model was Acrylonitrile Butadiene Styrene (ABS), a nonmagnetic material selected to eliminate any potential interference with magnetic measurements. The setup was designed with no direct contact with these walls to prevent the risk of generating magnetic fields inside the chamber—which can result from vibrations in the chamber walls. To further ensure that vibrations caused by the motor outside the chamber were not transferred to the chamber walls, we mounted an accelerometer (x-IMU3), on the chamber lids during the experiment. The x-IMU3, a third generation of Inertial Measurement Unit (IMU) device from x-io Technologies Limited, contains three types of sensors, including accelerometer, gyroscope, magnetometer. Results from accelerometer sensor showed negligible vibrational coupling from the moving arm to the chamber's walls.

### 2.4 Single channel tunnelling magnetoresistive sensor

All experiments were conducted with the chamber lids in place to protect against interference from the surrounding environment. Subsequently, the chamber was degaussed to ensure minimal residual magnetic fields remained inside and the residual background field was distributed homogeneously (29). The background magnetic field within the chamber was continuously monitored using an optically pumped magnetometer (OPM, Quspin, Inc.), verifying that the magnetic field generated matched our intended specification and that there was no external magnetic interference. Moreover, to eliminate any interference from frontend vibrations, the sensor was mounted on our 3D model. In contrast, the frontend was placed on a separate support inside the chamber, as shown in Figure 2A. This setup allowed us to assess the effect of vibrations on the sensor itself without interference from the frontend.

We have exposed a single-channel tunnelling-magnetoresistive (TMR) sensor developed by Neuranics Limited to constant vibration at a frequency of 8 Hz, oriented in the X direction, as illustrated in Figure 2B. Initially, a homogeneous DC field ranging from 10 nT to 100 nT was applied through coils installed within the chamber. Then, a gradient DC field ranging from 1 nT/mm to 10 nT/mm was introduced in the dz/dx direction to evaluate the impact of each condition on the vibration measurements. The data was captured and analysed in the frequency spectrum using a MFLI Lock-in Amplifier from Zurich Instruments.

### 2.5 Gradiometer

In this paper, we evaluate using a gradiometer as a potential solution to reduce vibration artifacts. A gradiometer, composed of separate magnetometers (two magnetometers, in this study), obtains a magnetic field gradient by subtracting the voltage signals (30, 31). To understand its performance and suitability for our needs, it is essential to characterise the gradiometer design.

**Figure 3:**
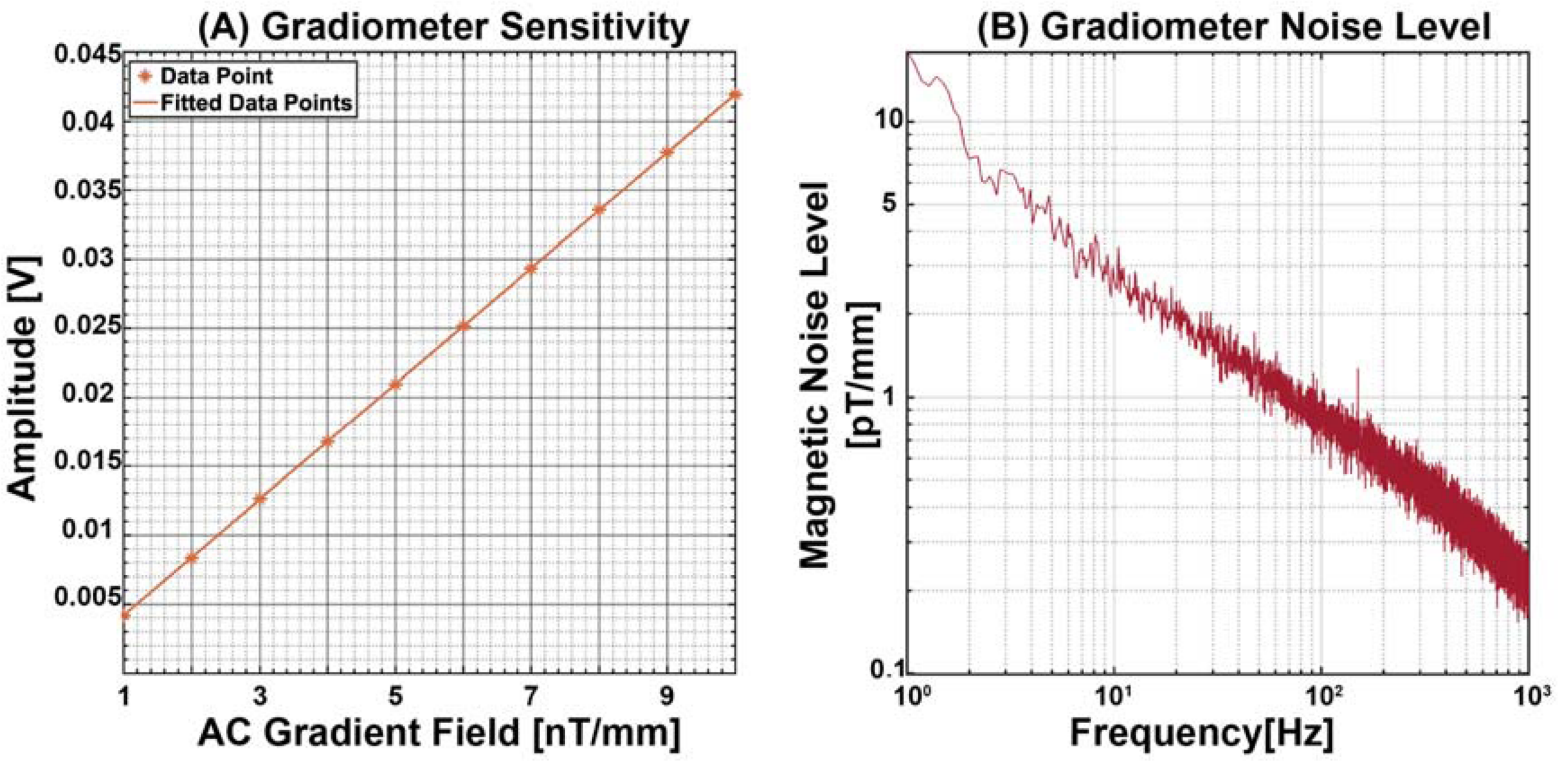
Gradiometer characterisation metrics. **(A)** Gradiometer sensitivity by applying a 10 Hz AC gradient field from 1 to 10 nT/mm. **(B)** Gradiometer noise level measurements show a 3.88 pT/mm at 8 Hz.

First, we measured the sensitivity of the gradiometer. This process involves applying an AC gradient field from 1 nT/mm to 10 nT/mm in the dz/dx axis (which was aligned with the gradient field applied in our experiment) at a frequency of 10 Hz (which was close to the frequency of our experiment, 8 Hz) and recording the corresponding output of the gradiometer. The sensitivity level was then calculated from this recorded output, which was 5.9311 mV/nT/mm, as shown in Figure 3A. Next, we assessed the voltage noise level of our system. To do this, we recorded the gradiometer output without applying any external input. This step is crucial as it helps us understand the intrinsic noise characteristics of the gradiometer itself, independent of any external influences. By dividing the measured voltage noise level by the previously determined sensitivity of the gradiometer, we obtain the gradiometer noise level. This parameter indicates the inherent noise performance of the gradiometer, which is demonstrated in Figure 3B.

For further characterisation, we used a metric called Common Mode Rejection Ratio (CMRR), commonly used in electronics, particularly for amplifiers. CMRR is the ratio between the output of the single channel and that of the differential channel (32), which measures the capability of reducing the common-mode magnetic signal between two channels. Hence, it can quantify the ability of the device to reject common-mode signals. A high CMRR suggests that the gradiometer can operate effectively in a noisy environment (33). To calculate the CMRR, we applied a 100 nT AC background magnetic field to the chamber at 10 Hz and then recorded the gradiometer and single channel output in this magnetic field. According to the equation below, the CMRR for our sensor is 75.9289 dB.

(1) $$\mathrm{CMRR}\;=\;20\;\mathrm{lo}g_{10}\left(\frac{\mathrm{Gradiometer}\;\mathrm{Output}}{\mathrm{Single}\;\mathrm{Channel}\;\mathrm{Output}}\right)\mathrm{dB}$$

After the gradiometer characterization, we followed the exact instructions for the single channel, applying a gradient DC magnetic field, 1 nT/mm to 10 nT/mm, to both the gradiometer and the single-channel sensor, and then compared the resulting data. We tested two configurations for the gradiometer: initially, a parallel configuration with the gradiometer baseline oriented in the X direction, followed by a vertical configuration with the baseline in the Y direction.

## 3 Results

### 3.1 Single channel output

**Figure 4:**
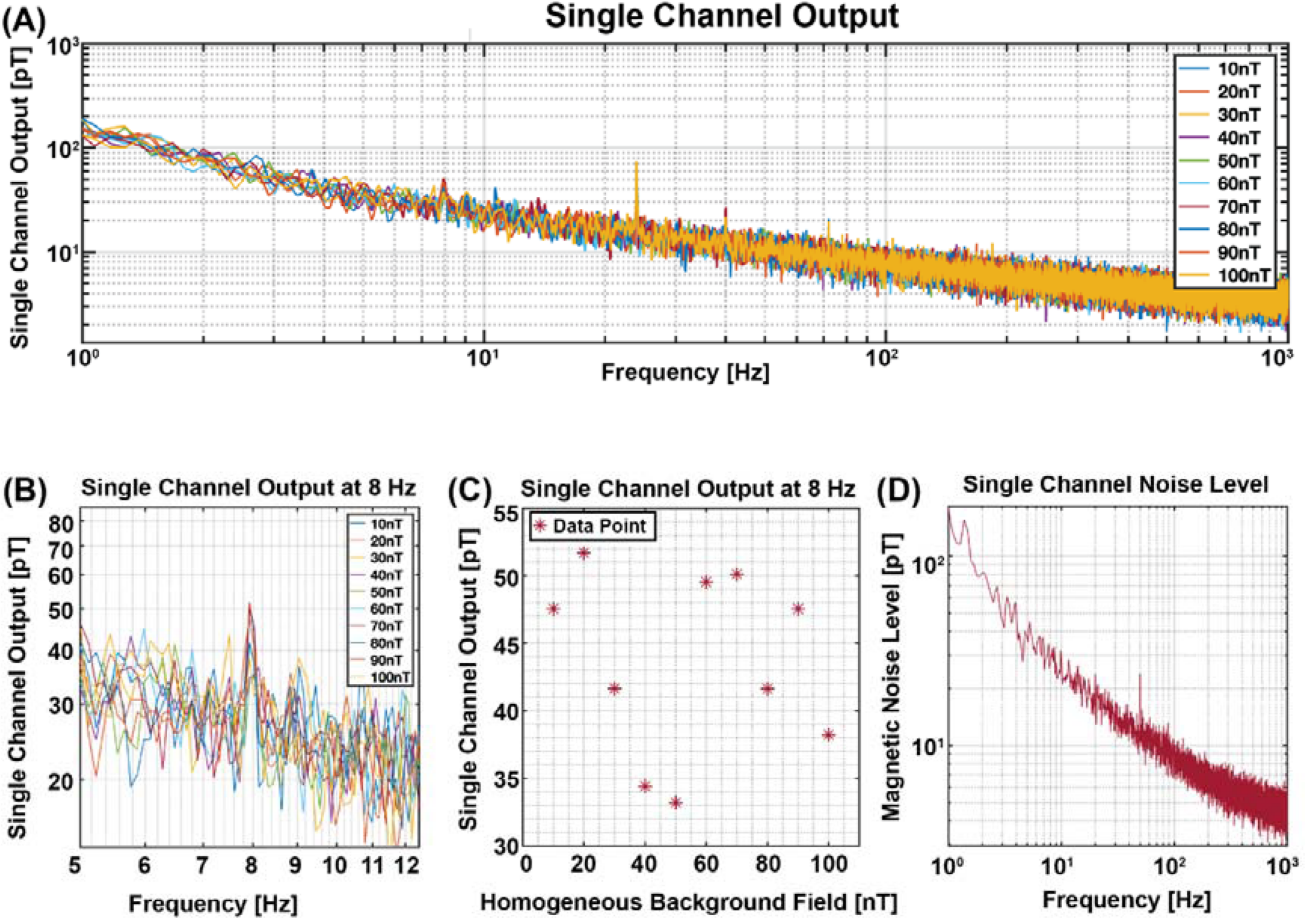
Single channel output in variable homogeneous background magnetic field. **(A)** Single channel output across the entire frequency bandwidth. **(B)** Zoomed-in view of the single channel output to enhance visibility at the 8 Hz vibration frequency. **(C)** Channel output at 8 Hz, illustrating no correlation between the signal output and the homogeneous background magnetic field, as the signal output is in the noise level. **(D)** Sensor noise level between 1 and 1,000 Hz frequency bandwidth, which is consistent with the signal output at 8 Hz in **(C)**.

While the sensor vibrated at a frequency of 8 Hz inside the magnetically shielded chamber, we applied a homogeneous background magnetic field ranging from 10 nT to 100 nT using coils implemented inside the chamber. The frequency spectrum response of the sensor is shown in Figure 4A, highlighting the 8 Hz frequency. For enhanced clarity, Figure 4B provides a zoomed-in view of Figure 4A, consistently displaying sensor outputs within the range of tens of picoteslas, which align with the expected noise level of the sensor, demonstrating negligible fluctuations. Determination of the peaks at the 8 Hz frequency, as illustrated in Figure 4C, revealed no discernible correlation between the sensor output and the background homogeneous magnetic field, as the sensor output consistently resided within the sensors magnetic noise level, as evidenced in Figure 4D. This indicates that the sensor's response to vibrations remains largely unaffected by variations in the homogeneous background magnetic field, as increasing the intensity of the homogeneous magnetic field did not significantly alter the sensor output.

**Figure 5:**
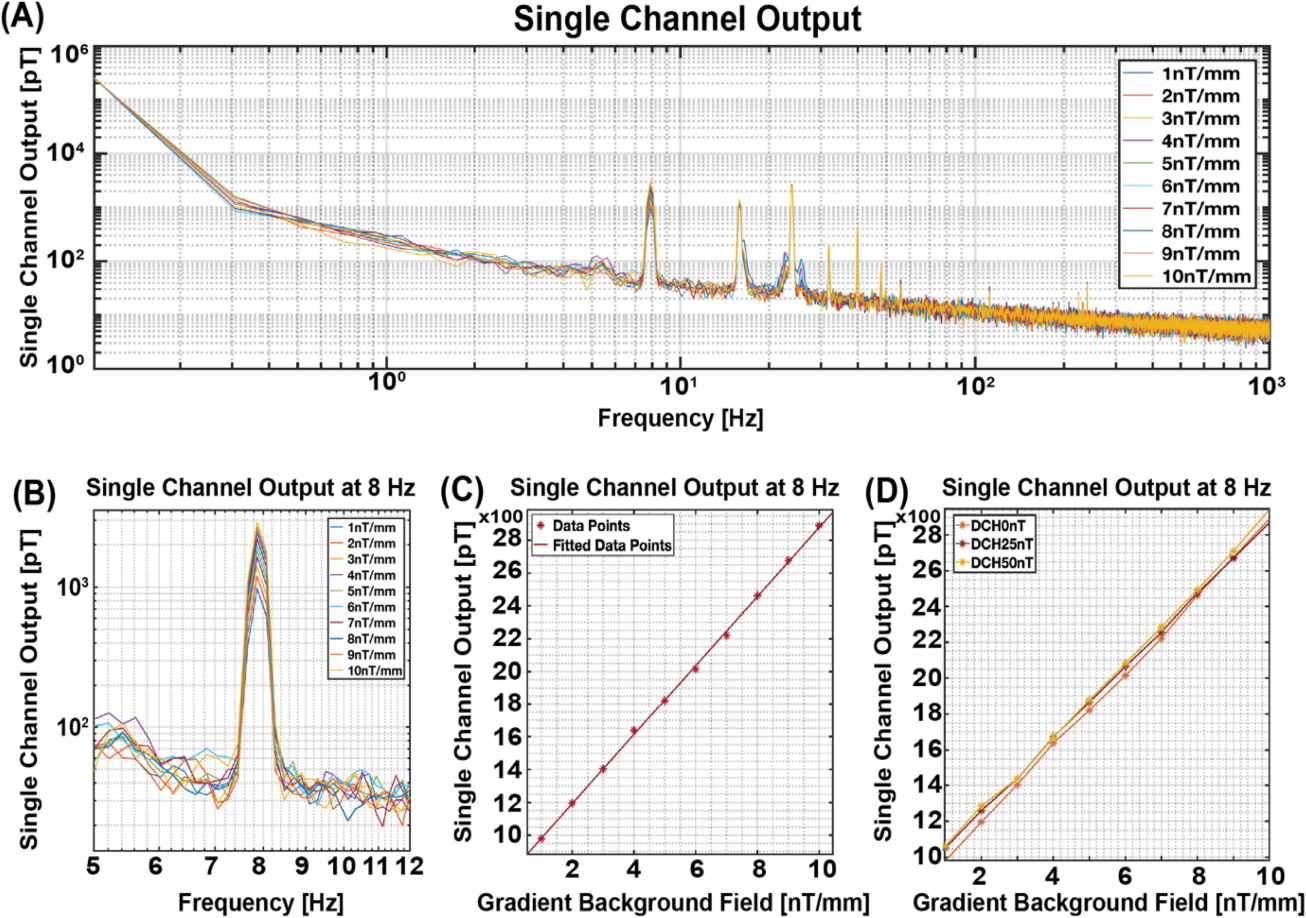
Single channel output in gradient background magnetic field. **(A)** Single channel output across the entire frequency bandwidth. **(B)** Zoomed-in view of the single channel output to enhance visibility at the 8 Hz vibration frequency. **(C)** Channel Output at 8 Hz, illustrating a slope of 210.70 pT for each unit increase in the background gradient magnetic field from 1 nT/mm to 10 nT/mm. **(D)** Fitted data points when adding gradient background magnetic field from 1 nT/mm to 10 nT/mm to three different DC homogeneous background fields (0, 25,50 nT).

Following the initial experiment, the sensor was subjected to vibration at a frequency of 8 Hz, within a gradient magnetic field ranging from 1 nT/mm to 10 nT/mm in dz/dx direction, while maintaining a constant homogeneous background magnetic field measured at approximately 0 nT. Figure 5B provides a zoomed-in perspective of Figure 5A, enhancing the observation at the 8 Hz vibration frequency. Remarkably, the results demonstrated a consistent increase in signal output, manifesting a slope of 210.79 pT for every unit increase in the gradient of the background magnetic field, as depicted in Figure 5C.

To further confirm the result, we repeated our experiment with varying levels of homogeneous background magnetic fields: 0 nT, 25 nT, and 50 nT, while maintaining the gradient background magnetic field at 1 nT/mm to 10 nT/mm. As shown in Figure 5D, the sensor output across these three homogeneous background magnetic fields follows a similar slope, indicative of a similar response despite differing baseline homogeneous background fields. This outcome confirms our hypothesis regarding the substantial impact of the gradient background magnetic field on the sensor's output, irrespective of fluctuations in the homogeneous background magnetic field.

### 3.2 Gradiometer output

**Figure 6:**
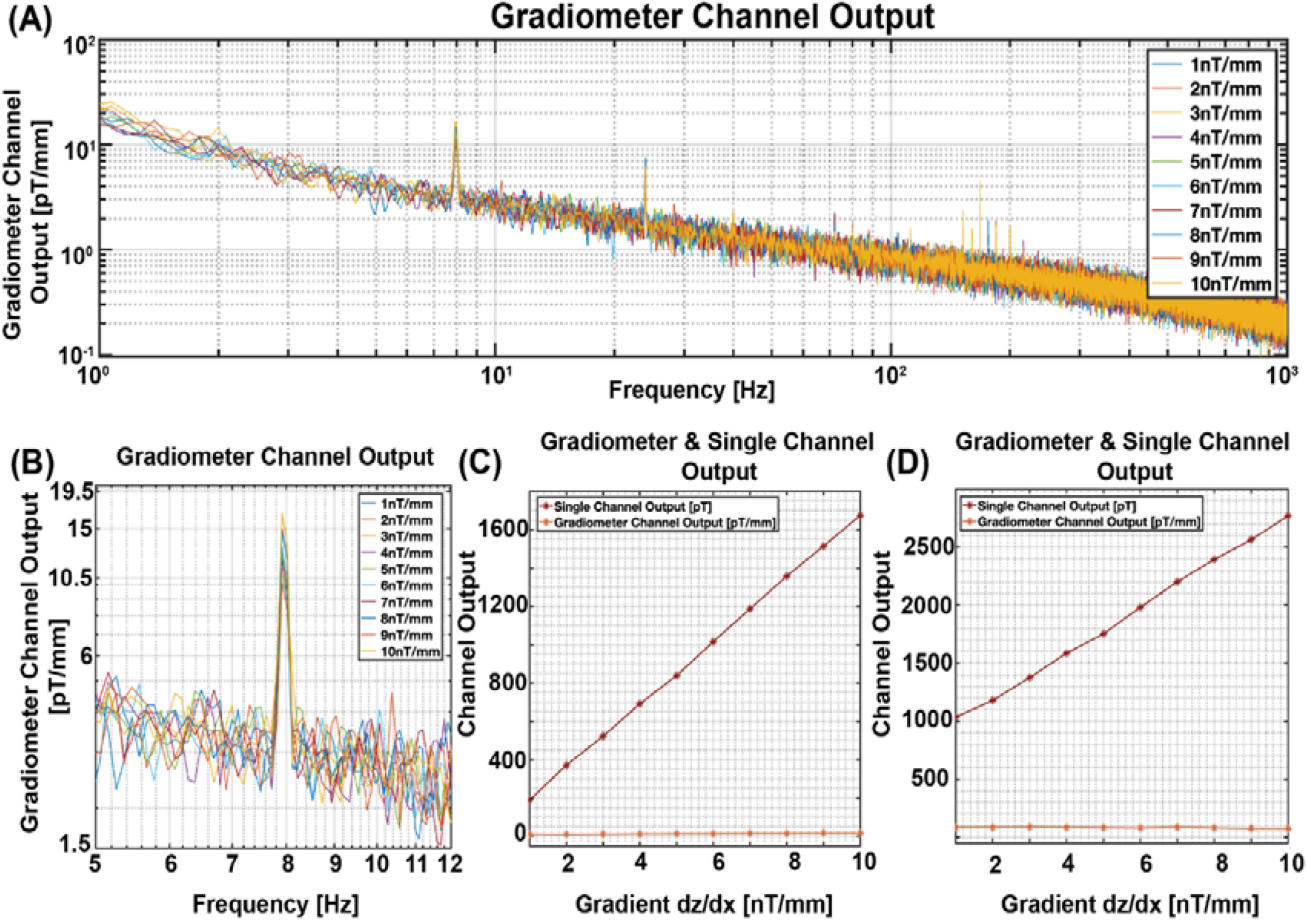
Gradiometer output in gradient background magnetic field. **(A)** Output of gradiometer with parallel configuration across the entire frequency bandwidth. **(B)** Zoomed-in view of the parallel gradiometer output to enhance visibility at the 8 Hz vibration frequency. **(C)** Parallel gradiometer configuration: Gradiometer Output at 8 Hz, illustrating a slope of 164.97 m^−1^ and 0.75 for single channel and gradiometer output, respectively, for each unit increase in the background gradient magnetic field ranging from 1 nT/mm to 10 nT/mm. **(D)** Vertical gradiometer configuration: Fitted data points at 8 Hz, illustrating a slope of 196.85 m^−1^ and 1.06 for single channel and gradiometer output, respectively, for each unit increase in the background gradient magnetic field ranging from 1 nT/mm to 10 nT/mm.

In this study, we demonstrated that the presence of a gradient magnetic field affects the sensor output when measuring motion or vibration artifacts. In contrast, the homogeneous background magnetic field exerts minimal influence on the output of the vibrating single-channel sensor. One proposed solution to eliminate the motion artifact is using a gradiometer. To assess the gradiometer's effect, we introduced a DC gradient magnetic field ranging from 1 nT/mm to 10 nT/mm in the dz/dx direction and a zero DC homogeneous magnetic field to a vibrating gradiometer setup in the X direction. Initially, the gradiometer was configured in a parallel arrangement, aligning its baseline with the direction of vibration (X-axis). Figure 6A depicts the entire frequency spectrum of the gradiometer response to the increased gradient background field. Upon closer examination in Figure 6B a zoomed-in view of Figure 6A, provides a more detailed perspective of the gradiometer output, indicating subtle changes in response to the increased gradient background field.

Then, the gradiometer and the single channel sensor outputs were recorded using the Lock-in Amplifier from Zurich Instruments, depicted in Figure 6C. Notably, the slope of the single channel sensor output derived from the gradiometer design measured 164.97 pT for each unit increase in the gradient of the background magnetic field. In contrast, the gradiometer output exhibited an increment of only 0.75 pT/mm for each stepwise increase in the gradient background magnetic field, spanning from 1 nT/mm to 10 nT/mm.

Subsequently, we repositioned the gradiometer in a vertical configuration, orienting its baseline in the Y direction, perpendicular to the vibration direction (X direction). As illustrated in Figure 6D, the slope of the single channel output of this gradiometer design measured 196.85 pT, representing a substantial 196.85 pT increment for every unit increase in the gradient background magnetic field within the 1 nT/mm to 10 nT/mm range. However, the corresponding increase in the gradiometer output was 1.06 pT/mm for each incremental rise in the gradient background magnetic field.

## 4 Discussion

This study reveals that, while increasing the homogeneous background magnetic field does not affect the sensor output, increasing the gradient background magnetic field significantly enhances the sensor output. To confirm this hypothesis, we repeated the experiment by adding a gradient ranging from 1 nT/mm to 10 nT/mm to different homogeneous background fields. The slope for the sensor output remained similar, highlighting the substantial effect of the gradient background magnetic field compared to the homogeneous one. These findings demonstrate why movement artifacts are more pronounced and impactful in typical ambient environments, where electrical equipment and other sources generate a gradient magnetic field. Additionally, for biomagnetic measurements inside a magnetically shielded chamber or room, attention should be focused on the residual gradient background magnetic field rather than the homogeneous background field.

This paper only assessed linear vibrations, whereas in the real-world, more complex rotational vibrations influence the magnetic measurements (34, 35). For example, a sensor placed on the arm will not only vibrates in the x, y, and z axes but also will experience changes in pitch and yaw angles. Rotational motion is beyond the scope of this work because, firstly, it would be hard to simulate it off-body, and secondly, due to the complex/non-repeatability nature of angles for the same body movement.

One proposed solution to eliminate the effect of linear vibration is using a gradiometer. By positioning the gradiometer in two configurations, parallel and vertical, to the vibrational axis, we observed a subtle change in the gradiometer output compared to the single-channel output. These findings essentially indicate that a gradiometer can effectively mitigate the impact of motion artifacts in ambient environments where existing gradient magnetic fields affect the output of a single sensor, potentially masking the desired recorded signals. Moreover, this mitigation remains effective even when the gradiometer baseline is not aligned with the direction of motion.

To summarise, the TMR sensor measurements will be influenced by movement artifacts, different types of background magnetic fields: Homogeneous DC magnetic field (HDC), Homogeneous AC magnetic field (HAC), Gradient DC magnetic field (GDC) and Gradient AC magnetic field (GAC). These movement artifacts can be aligned with or perpendicular to the gradient. We consider two configurations for the gradiometer: Parallel Configuration (the baseline is aligned with the movement artifact) and Vertical Configuration (gradiometer's baseline is perpendicular to the movement artifact).

As shown in Tables 1, 2, the gradiometer is suitable for rejecting both homogeneous and gradient DC fields. The analysis assumed that the gradient is linearly distributed across the space. For a more general non-linear gradient field, we can simplify these tables as the mathematic equation mentioned in Equation 2:

(2) $$B_{gradio}\;=\;\delta B\;\times \;BL$$

where the δB is the gradient vector of the magnetic field and BL is the baseline vector of the gradiometer. Any movement that changes δB will result in the final output of the gradiometer.

**Table 1:** Ac coupled single TMR sensor; sensor output will be influenced by.

	Movement aligned with gradient	Movement perpendicular to gradient
HDC	✕	✕
HAC	✓	✓
GDC	✓	✕
GAC	✓	✕

**Table 2:** Ac coupled gradiometer; gradiometer output will be influenced by.

		Movement aligned with gradient	Movement perpendicular to gradient
HDC		✕	✕
HAC		✕	✕
GDC	Parallel	✕	✕
	Vertical	✕	✕
GAC	Parallel	✓	✕
	Vertical	✓	✓

Therefore, as we move towards wearable devices, where separation between the sensor and the skin source is not feasible, we can use gradiometer to reduce the recording of artifacts that lie in the lower frequency bandwidth and cannot be eliminated by simple filtering.

## 5 Conclusion

This paper demonstrated that the gradient magnetic field significantly affects the single-channel sensor output, while the homogeneous field has minimal impact. To simulate motion artifacts, a magnetised linear motor generated controlled vibratory motion at 8 Hz, transferred to the chamber through a 3D-printed setup. By altering the background magnetic field using implemented coils inside the chamber, we investigated the effect of each condition.

Our measurements indicated a negligible change in the single-channel output with an increase in the homogeneous background magnetic field in the 10–100 nT range. However, the single-channel sensor output exhibited a significant change of 164.97 pT per unit increase in the gradient background magnetic field within the range of 1 nT/mm to 10 nT/mm. When following the same procedure for the gradiometer in the parallel alignment with the vibration direction, the gradiometer output only changed by 0.75. Then, when the gradiometer was repositioned vertically (Y direction), perpendicular to the vibration direction, the single-channel output slope increased to 196.85 m^−1^, whereas the gradiometer output only increased by 1.06 for the same gradient range. These results highlight the significant influence of gradient magnetic fields on motion artifacts and demonstrate the effectiveness of gradiometers in mitigating these effects. Therefore, as gradiometers are expected to be suitable for wearable biomagnetic applications, their future implementations in wearable systems will enable real-time cancellation of motion artifacts, enhancing the accuracy and reliability of biomagnetic measurements.

## Data Availability

The raw data supporting the conclusions of this article will be made available by the authors, without undue reservation.
